# IL-10 Indirectly Downregulates IL-4–Induced IgE Production by Human B Cells

**DOI:** 10.4049/immunohorizons.1800076

**Published:** 2018-12-18

**Authors:** Adora A. Lin, Alexandra F. Freeman, Thomas B. Nutman

**Affiliations:** * Helminth Immunology Section, Laboratory of Parasitic Diseases, National Institute of Allergy and Infectious Diseases, National Institutes of Health, Bethesda, MD 20892; † Laboratory of Clinical Immunology and Microbiology, National Institute of Allergy and Infectious Diseases, National Institutes of Health, Bethesda, MD 20892

## Abstract

Controlled allergic disease is associated with decreased allergen-specific IgE and increased allergen-specific IgG4. Although IL-10 has been shown to contribute to these changes, the underlying mechanisms are largely unknown. This study explored how IL-10 differentially regulates human IgE and IgG4 production. Highly purified B cells and B cell subsets from healthy individuals were cultured with combinations of anti-CD40, IL-4, and IL-10. In other experiments, PBMCs isolated from healthy donors or from autosomal dominant hyper-IgE syndrome (STAT3 loss-of-function) subjects were cultured with combinations of IL-4 and IL-10. In B cell cultures, IL-10 had no significant effect on IL-4–induced IgE production but increased IL-4–induced IgG4 production over 20-fold. IL-4-induced transcription of Cε and Cγ4 germline transcripts (GLTs) by isolated B cells was not affected by IL-10. In PBMC cultures, IL-4 induced production of both IgE and IgG4 and increased expression of Cε and Cγ4 GLTs above baseline. Unlike in purified B cells, IL-10 diminished IL-4–induced IgE production and expression of Cε GLTs without affecting IgG4 production or expression of Cγ4 GLTs. PBMCs from autosomal dominant hyper-IgE syndrome individuals failed to consistently modulate IgE production in response to IL-4 and IL-10. As measured by flow cytometry, the frequency of IL-10R^+^ cells was similar between IgE^+^ and IgG4^+^ B cells. These data suggest that IL-10 acts indirectly through accessory cells to modulate the production of IgE. For IgG4, IL-10 appears to act directly on B cells to drive IgG4 production, with its effects being downstream of germline transcription.

## INTRODUCTION

Controlled allergic disease is associated with decreased levels of allergen-specific IgE and increased levels of allergen-specific IgG4. Following allergen immunotherapy, allergen-specific IgE levels are decreased, with a concomitant increase in allergen-specific IgG4 compared with preimmunotherapy levels (1, 2). Subjects tolerant to high doses of allergen, such as beekeepers or cat owners, also have higher levels of allergen-specific IgG4 compared with allergic individuals (2–4), suggesting that an increased IgG4/IgE ratio may modulate allergic effector responses.

Class switch to IgE requires two signals, the first provided by the interaction of CD40 on the B cell with CD40L on T cells and other cells (5–7). The second signal comes from the cytokines IL-4 and IL-13 (5–7). Many cytokines are able to augment IL-4– or IL-13-induced IgE production, including IL-5, IL-6, IL-9, and TNF-α (8–12). Other factors, including PGE_2_, IFN-α, IFN-γ, and TGF-β, are able to downregulate IgE production (9,13). IgG4 class switching and production are modulated by the same cytokines (5, 7, 14), making selective regulation of these isotypes for potential therapeutics difficult.

IL-10 has been shown to decrease isotype switch to, and production of, IgE while promoting IgG4 production from PBMCs (14, 15). This suggests that IL-10 may differentialy regulate these two isotypes, as has been shown with IL-12 (16), IL-21 (17), and factors produced by filarial-activated B cells (18). Frequencies of IL-10–producing T and B cells are increased following allergen immunotherapy (19–21), suggesting that IL-10 maybe the relevant cytokine regulating IgE and/or IgG4 levels during the development of allergic tolerance.

Little is known about the mechanisms by which IL-10 might mediate these differential effects on IgE and IgG4 production. Upon the binding of IL-10 to IL-10R, the Janus kinases Jak1 and Tyk2 associate with the receptor and aid in the recruitment of STAT3 (22). Dominant-negative, loss-of-function mutations in STAT3 in humans leads to autosomal dominant hyper-IgE syndrome (HIES) (AD-HIES), also known as Job syndrome (23–27). These patients display recurrent pulmonary and skin infections, chronic dermatitis, and elevated serum IgE levels, suggesting that STAT3 signaling is also important for regulation of IgE production.

This study explored how IL-10 regulates IgE and IgG4 production using cultures of human PBMCs and highly purified peripheral B cells. Our data show that IL-10 indirectly downregulates IgE production through accessory cells present in PBMCs. Concurrently, IL-10 acts directly on Ag-experienced B cells to drive IgG4 production. These findings have important implications for new therapeutic approaches to allergic diseases and other diseases in which IgE production is dysregulated.

## MATERIALS AND METHODS

### Clinical samples

Buffy coats and whole-blood samples were obtained from healthy adult donors as part of a protocol from the Department of Transfusion Medicine, Clinical Center, National Institutes of Health (Institutional Review Board no. 99-CC-0168) for the healthy donors. Anticoagulated whole blood from patients with AD-HIES with loss-of-function STAT3 mutations was obtained as part of a National Institute of Allergy and Infectious Diseases Institutional Review Board-approved registered trial (NCT00006150). Written informed consent was obtained from all subjects. Samples used were both fresh and cryopreserved.

### Cell preparations

PBMCs were isolated from buffy coats by density gradient centrifugation (LSM Lymphocyte Separation Medium; MP Biomedicals, Santa Ana, CA), and RBCs were removed by hypotonic lysis (ACK Lysing Buffer; Life Technologies, Gaithersburg, MD). B cells were purified from PBMCs (following RBC lysis and washing) by negative selection using magnetic beads (EasySep Human B Cell Enrichment Kit with addition of EasySep Human CD10 Positive Selection Cocktail [STEMCELL Technologies, Cambridge, MA]). B cells were further fractionated into CD27^+^ and CD27^−^ subsets by incubating with biotinylated anti-CD27 Ab (clone 0323; Thermo Fisher Scientific [TFS], Waltham, MA) and Anti-Biotin MicroBeads (Miltenyi Biotec [MB], Auburn, CA) prior to separation on an autoMACS Pro (MB). Resultant cell populations were checked for purity by flow cytometric analysis. B cells were routinely ~98% pure and contained <0.05% CD3^+^ cells. Isolated CD27^+^ B cells were routinely >90% pure, and isolated CD27^−^ B cells were routinely ~99% pure and contained <0.5% CD27^+^ B cells.

### Abs and flow cytometry

For evaluation of purity, cells were stained in 96-well plates with fluorescently labeled Abs specific for CD3 (clone UCHT1; BD Biosciences [BD], San Jose, CA), CD19 (clone SJ25C1; TFS), CD21 (clone HB5; TFS), and CD27 (clone L128; BD). For evaluation of IL-10R expression, cells were stained with fluorescently labeled Abs specific for CD3, CD19, CD20 (clone 2H7; TFS), CD24 (clone SN3 A5–2H10; TFS), CD27 (clone 0323; TFS), CD38 (clone HIT2; TFS), IgD (clone IA6–2; TFS), and IL-10R (clone 3F9; BioLegend, San Diego, CA). All Abs were titrated prior to use in experiments. Cells were stained in the presence of Human FcR Blocking Reagent (MB) and fixed with 2% paraformaldehyde prior to analysis. The gating strategy for B cell populations is shown in Supplemental Fig. 1.

For intracellular Ig staining, cells were stained with fluorescently labeled Abs specific for CD3, CD19, IgD, CD27, CD38, FceRI (clone AER-37; TFS), and IL-10R in the presence of Human FcR Blocking Reagent. Samples were then fixed and permeabilized using the Foxp3/Transcription Factor Staining Buffer Set (TFS) according to the manufacturer’s directions. Cells were then stained with fluorescently labeled Abs specific for IgE (clone Ige21; TFS) and IgG4 (clone HP6025; SouthernBiotech, Birmingham, AL). Subsequent washes were done with 1× Permeabilization Buffer (TFS) according to the manufacturer’s directions and resuspended in PBS plus 2% heat-inactivated FBS (Gemini Bio-Products, West Sacramento, CA) prior to data acquisition. The gating strategy for IgE^+^ and IgG4^+^ B cells is shown in Supplemental Fig. 2.

Data were acquired on an LSR II or LSRFortessa (BD) and analyzed using FlowJo software (FlowJo, Ashland, OR).

### Cell culture

PBMCs were cultured at 2 × 10^5^ cells/well in a volume of 200 μl in 96-well, round-bottom plates. B cells were cultured at 3 × 10^5^ cells/well in a volume of 200 μl in 96-well, flat-bottom plates. Cells were cultured in Yssel’s Serum-Free T-Cell Medium (Gemini BioProducts) with 10% heat-inactivated FBS (Gemini Bio-Products), 2 mM L-glutamine (Life Technologies), and penicillin/streptomycin (Life Technologies). Cells were stimulated with combinations of recombinant human IL-4 (40 ng/ml) and IL-10 (50 ng/ml; PeproTech, Rocky Hill, NJ). For B cell cultures, 0.1 μg/ml ofanti- CD40 Ab (clone 82111; R&D Systems, Minneapolis, MN) was added.

### Germline transcript PCR

Cells were harvested on day 4 of culture. RNA was isolated using the RNeasy Mini Kit (Qiagen, Germantown, MD) according to the manufacturer’s directions. cDNA was synthesized using qScript cDNA SuperMix (Quanta BioSciences, Beverly, MA), also according to manufacturer’s directions. Quantitative PCR was performed using primers and probes specific for germline Cε and Cγ4 transcripts (Applied Biosystems/TFS). Primer and probe sequences are as follows: Cε forward, 5′-CATCCACAGGCAC CAAATGG-3′; Cε reverse, 5′-GACGGATGGGCTCTGTGT-3′; Cε probe, 6-fluorescein-ACCCGGCGCTTCAG-minor groove binder-nonfluorescent quencher; Cγ4 forward, 5’-GGCCGGCAG CATCAC-3’; Cγ4 reverse, 5’-CCGATGGGCCCTTGGT-3′; and Cγ4 probe, 6-fluorescein-AAGGACAGCAGCTTCC-minor groove binder-nonfluorescent quencher. PCRs were prepared with TaqMan Fast Advanced Master Mix 2 × buffer (TFS) with a final volume of 10 ml. Cε and Cγ4 germline transcript (GLT) primers were used at a final concentration of 900 nM, and probes were used at a final concentration of 250 nM. Each reaction was performed in quadruplicate. Usingthe ViiA 7 Real-Time PCR System (TFS), an initial 20-s incubation at 95°C was followed by two-step PCR cycling between 95°C for 1 s and 60°C for 20 s for 40 cycles. Samples were normalized to the expression of the 18S ribosomal gene using the Eukaryotic 18S rRNA Endogenous Control primer/probe mixture (TFS).

### Ig assays

Cell culture supernatants were harvested on day 12 to quantify Ig. Each of the Ab isotypes were quantified using the Bio-Plex Pro Human Isotyping Panel, 6-plex, and Human IgE Isotyping Assay (Bio-Rad Laboratories, Hercules, CA), according to the manufacturer’s directions. Samples were run on a Bio-Plex MAGPIX Multiplex Reader (Bio-Rad Laboratories).

### Statistical analyses

Unless otherwise stated, geometric means (GMs) were used as a measure of central tendency. Wilcoxon matched pairs signed-rank test was used to compare responses at the individual donor level. Graphs and statistical analyses were generated with GraphPad Prism Software (version 7; GraphPad Software, La Jolla, CA).

## RESULTS

### IL-10 significantly upregulates IL-4–induced production of IgG4 by B cells

**FIGURE 1.**
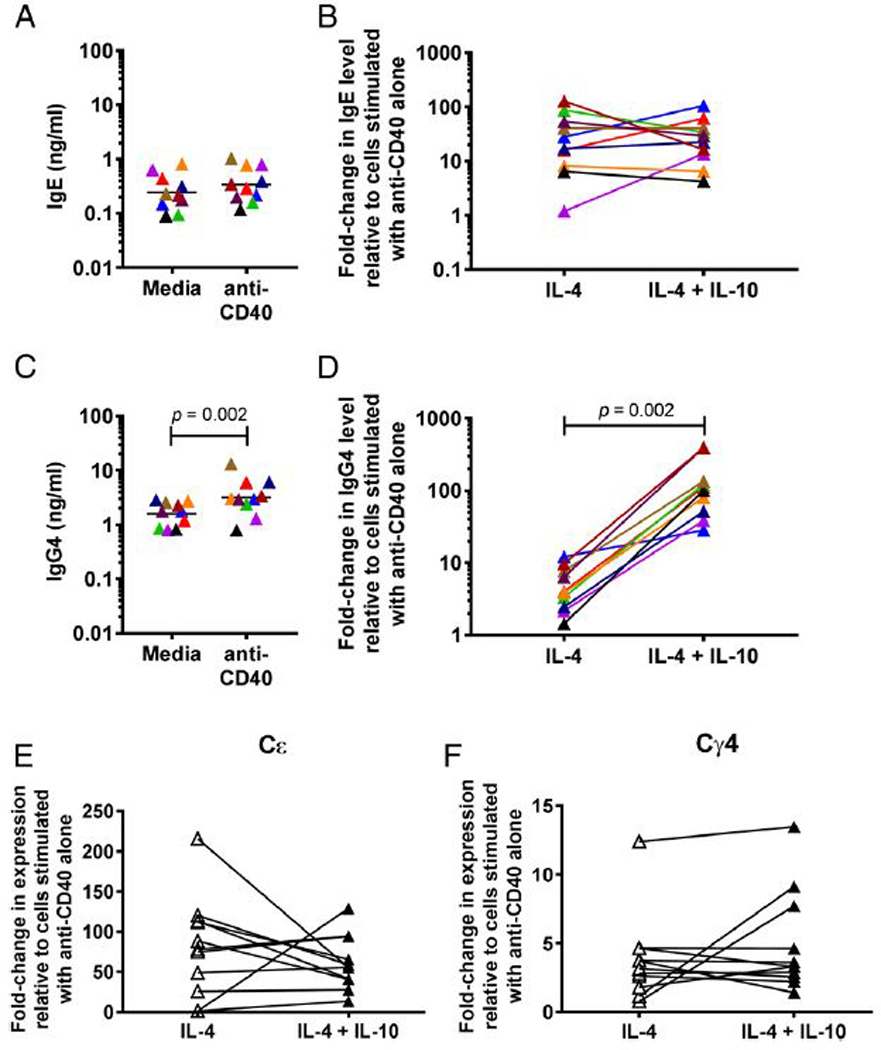
IL-10 upregulates IL-4-induced IgG4 production from B cells downstream of GLT expression. (**A** and **C**) IgE (A) and IgG4 (C) in culture supernatants of isolated total B cells at baseline and following addition of anti-CD40. (**B** and **D**) Fold change in levels of IgE (B) and IgG4 (D) in culture supernatants of isolated total B cells stimulated with anti-CD40 and IL-4 with and without IL-10, relative to B cells cultured with anti-CD40 Ab alone (*n* = 10). Each individual is represented with a unique color; colors are consistent for (A)–(D). (**E** and **F**) Fold change in expression of Cε (E) and Cγ4 (F) GLTs in isolated total B cells stimulated with anti-CD40 Ab and IL-4 with (filled triangles) and without (open triangles) IL-10 relative to B cells stimulated with anti-CD40 Ab alone (*n* = 11). The *p* values were determined using Wilcoxon matched pairs signed-rank test.

Previous studies have shown that IL-10 negatively regulates IL-4–induced IgE production and upregulates IL-4-induced IgG4 production in PBMCs (14, 15). To see if this was a direct effect on B cells, we isolated B cells from 10 healthy donors and stimulated them with anti-CD40 Ab, IL-4, and IL-10. Spontaneous IgE production from the isolated B cells ranged from 0.09 ng/ml to 0.81 ng/ml (GM of 0.25 ng/ml; Fig. 1A); spontaneous IgG4 production ranged from 0.80 ng/ml to 2.87 ng/ml (GM of 1.58 ng/ml; Fig. 1C). Production of IgE did not significantly increase with anti-CD40 stimulation alone, whereas production of IgG4 increased 2-fold (GM of 3.17 ng/ml) following anti-CD40 stimulation (*p* = 0.002; Fig. 1C). Anti-CD40 and IL-4 stimulation induced a GM 20-fold increase in IgE and a GM 4-fold increase in IgG4 production above production with anti-CD40 alone (Fig. 1B, 1D). The addition of IL-10 did not significantly alter IgE production (Fig. 1B). In contrast, IL-10 dramatically increased IL-4-induced IgG4 production 80-fold compared with stimulation with anti- CD40 alone (Fig. 1D).

IL-10 has been shown to affect class switching to IgE and IgG4 in PBMCs, as evidenced by decreased IL-4–induced expression of Cε GLTs in the presence of IL-10 (14, 15). Using isolated B cells, anti-CD40 plus IL-4 stimulation was able to induce the GLT expression of Cε and Cγ4 by ~ 40-fold and 3-fold, respectively (Fig. 1E, 1F). The addition of IL-10 had no significant effect on either Cε or Cγ4 GLT (Fig. 1E, 1F).

**FIGURE 2.**
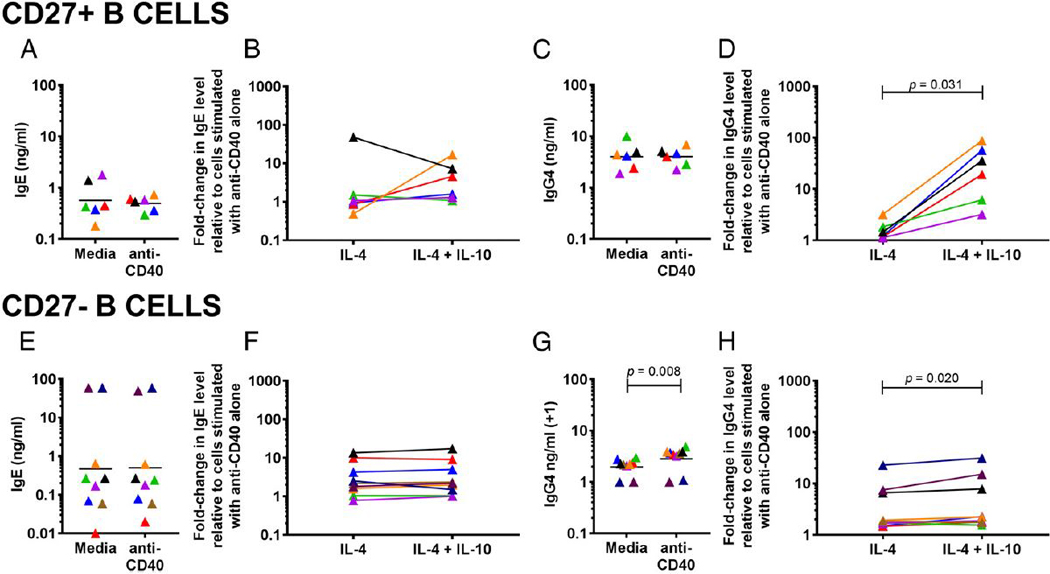
IL-10 upregulates IgG4 production by CD27^+^ B cells. (**A** and **C**) IgE (A) and IgG4 (C) in culture supernatants of isolated CD27^***+***^ B cells at baseline and following addition of anti-CD40. (**B** and **D**) Fold change in levels of IgE (B) and IgG4 (D) in culture supernatants of isolated CD27^+^ B cells stimulated with anti-CD40 Ab and IL-4 with and without IL-10 relative to CD27^+^ B cells cultured with anti-CD40 Ab alone (*n* = 6). (**E** and **G**) IgE (E) and IgG4 (G) in culture supernatants of isolated CD27^−^ B cells at baseline and following addition of anti-CD40. (**F** and **H**), Fold change in levels of IgGE (F) and IgG (H) in culture supernatants of isolated CD27^−^ B cells stimulated with anti-CD40 Ab and IL-4 with and without IL-10 relative to B cells cultured with anti-CD40 Ab alone (*n* = 9). Each individual is represented with a unique color; colors are consistent for (A)–(H). The *p* values were determined using Wilcoxon matched pairs signed-rank test.

To determine whether IL-10 was altering IgG4 production by newly switched, Ag-naive B cells (CD27^−^) or by Ag-experienced, previously switched memory B cells (CD27^+^), B cells were fractionated into CD27^+^ and CD27^−^ populations before stimulation with anti-CD40 Ab and IL-4 with or without IL-10. In CD27^+^ B cells, spontaneous production of IgE ranged from 0.18 ng/ml to 1.78 ng/ml (GM of 0.57 ng/ml; Fig. 2A), whereas spontaneous production of IgG4 ranged from 1.90 ng/ml to 10.17 ng/ml (GM of 4.02 ng/ml; Fig. 2C). Production of neither IgE nor IgG4 was significantly affected by the addition of anti-CD40 alone (Fig. 2A, 2C). Addition of anti-CD40 and IL-4 induced IgE production by CD27^+^ B cells slightly, but not significantly, above production with anti-CD40 alone (GM 1.77-fold increase; *p* = 0.844; Fig. 2B). IgG4 production by CD27^+^ B cells was also slightly increased above production with anti-CD40 alone (GM 1.55-fold increase; *p* = 0.031; Fig. 2D). IL-10 had no significant effect on IL-4–induced IgE production in CD27^+^ B cells, but IgG4 production was markedly increased by 1300% to a GM 20-fold above production with anti-CD40 alone (Fig. 2D).

In CD27^−^ B cells, spontaneous production of IgE ranged from 0.01 ng/ml to 60.0 ng/ml (GM of 0.47 ng/ml) and was unchanged by anti-CD40 (Fig. 2E). Spontaneous production of IgG4 ranged from undetectable levels to 2.0 ng/ml (GM of 0.97 ng/ml), with a slight increase in the presence of anti-CD40 (GM of 1.83; *p* = 0.008; Fig. 2G). Stimulation with anti-CD40 and IL-4 increased both IgE and IgG4 production to a GM ~3-fold above production with anti-CD40 alone (Fig. 2F, 2H). Similarly to CD27^+^ B cells, IL-10 had no effect on IL-4−induced IgE production in CD27^−^ B cells (Fig. 2F). IgG4 production was slightly, but significantly, increased in the presence of IL-10 (Fig. 2H).

### IgE^+^ B cells express IL-10R

**FIGURE 3.**
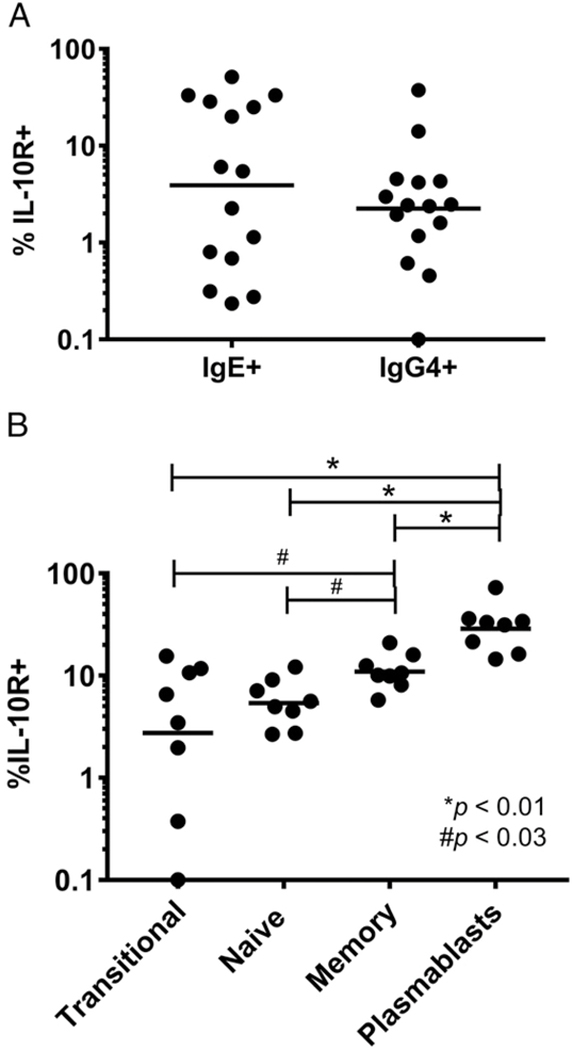
The IL-10R on both IgE^+^ and IgG4^+^ B cells and all B cell subsets. (**A**) Frequency of IL-10R^+^IgE^+^ and IgG4^+^ B cells. Dots represent individual patients. Lines indicate the GM for each cell population (*n* = 15). (**B**) Frequency of IL-10R^+^ cells in transitional, naive, memory, and plasmablast B cells. Surface markers used to define these subsets are in the text (*n* = 9). The *p* values were determined using Wilcoxon matched pairs signed-rank test. *p < 0.01, #*p* < 0.03.

Given the lack of an effect of IL-10 on IL-4–induced IgE production, we assessed whether IgE-producing B cells were capable of responding to IL-10 (i.e., whether they expressed IL-10R). PBMCs from healthy donors were stained for IL-10R and stained intracellularly for IgE and IgG4. Using intracellular staining, ~0.3% of CD19^+^ B cells were IgE^+^, and ~0.4% of CD19^+^ B cells were IgG4^+^ (Supplemental Fig. 2); these frequencies were similar to those seen in other studies using surface-only staining (28). Both IgE^+^ and IgG4^+^ B cells (CD3~FcεRI~CD19^+^IgD^−^) expressed the IL-10R at low frequencies (GM of 3.91 and 2.25%, respectively). There were no significant differences in the frequencies of IL-10R^+^ cells between IgE^+^ and IgG4^+^ B cells (Fig. 3A).

We also assessed the frequency of IL-10R expression on B cell subsets to see which types of B cells have increased capacity to respond to IL-10. PMBCs from healthy donors were stained for IL-10R and markers for transitional B cells (CD19^+^CD24^+^CD38^+^), naive B cells (CD19^+^CD27^−^IgD^+^), memory B cells (CD19^+^CD27^+^IgD^−^), and plasmablasts (CD19^+^CD24^−^CD38^+^). All subsets contained IL-10R^+^ cells. IL-10R was most frequently expressed on plasmablasts (GM of 28.78%), followed by memory B cells (GM of 10.96%), then naive (GM of 5.39%) and transitional B cells (GM of 2.75%) (Fig. 3B).

### IL-10 significantly decreases IL-4-induced production of IgE by PBMCs

**FIGURE 4.**
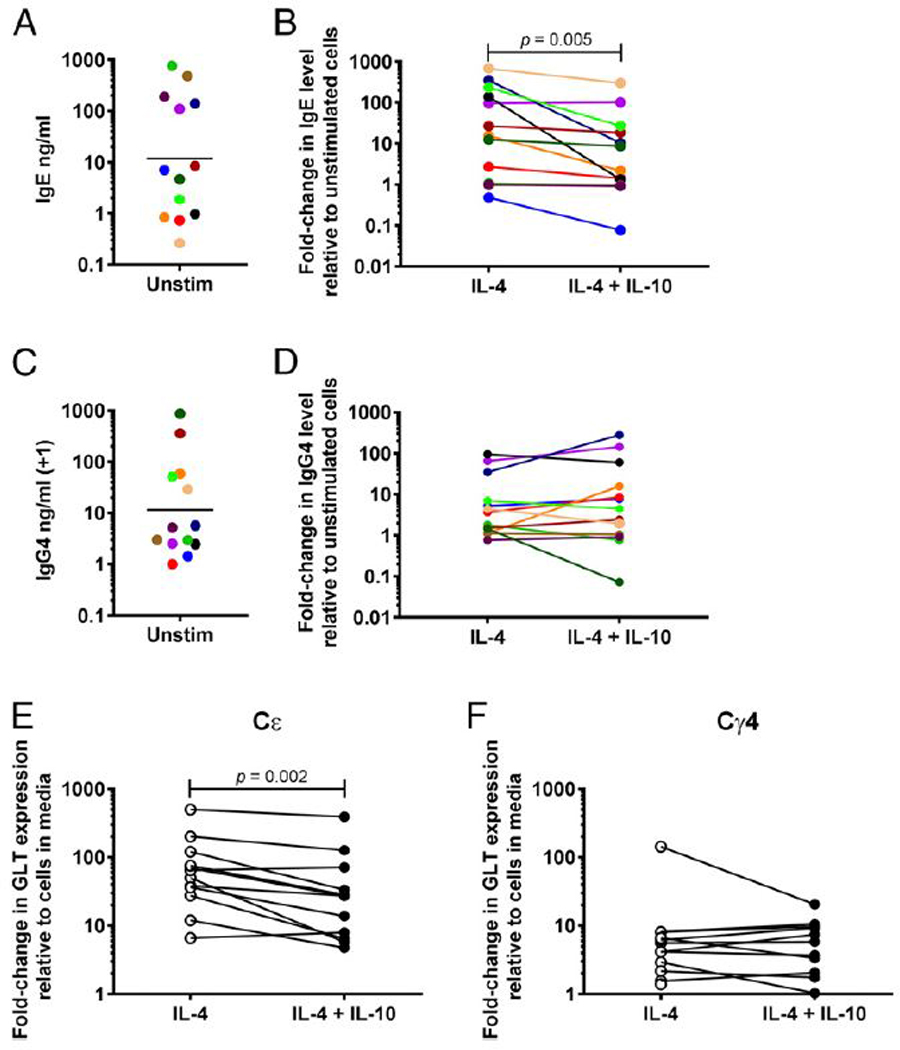
IL-10 decreases IL-4-induced expression and production of IgE in PBMC cultures. (**A** and **C**) Spontaneous production of IgE (A) and IgG4 (C) by PBMCs. (**B** and **D**) Fold change in levels of IgE (B) and IgG4 (D) in culture supernatants of PBMCs stimulated with IL-4 with and without IL-10 relative to PBMCs cultured in media alone (*n* = 13). Each individual is represented with a unique color; colors are consistent for figures (A)–(D). (**E** and **F**) Fold change in expression of Cε (E) and Cγ4 (F) GLTs in PBMCs stimulated with IL-4 with (closed circles) and without (open circles) IL-10 relative to PBMCs cultured in media alone (*n* = 12). The *p* values were determined using Wilcoxon matched pairs signed-rank test. Unstim, unstimulated.

We next examined whether IL-10 could act indirectly through accessory cells present in PBMCs to alter IgE and IgG4 production. PBMCs from healthy donors were stimulated with IL-4 with and without IL-10. Spontaneous production of IgE ranged from 0.27 ng/ml to 770.0 ng/ml (GM of 11.85 ng/ml; Fig. 4A). Spontaneous production of IgG4 ranged from undetectable to 870.3 ng/ml (GM of 10.54 ng/ml; Fig. 4C). Production of IgE and IgG4 with IL-10 alone was not significantly different from spontaneous production (data not shown). IL-4 induced production of both IgE and IgG4 to GMs of 16.22- and 4.68-fold over baseline, respectively (Fig. 4B, 4D). IL-4 also increased expression of Cε and Cγ4 GLTs to GMs of 54-fold and 5-fold above baseline, respectively (Fig. 4E, 4F). Addition of IL-10 significantly reduced IL-4–induced IgE production by ~72% to a GM of 4.57-fold above baseline; expression of Cε GLTs was also significantly decreased over 50% to 25-fold compared with baseline (Fig. 4B, 4E). In contrast, IL-10 did not significantly affect IgG4 production or expression of Cγ4 GLTs (Fig. 4D, 4F).

### IL-4 and IL-10 fail to consistently modulate IgE production by PBMCs from individuals with AD-HIES

**FIGURE 5.**
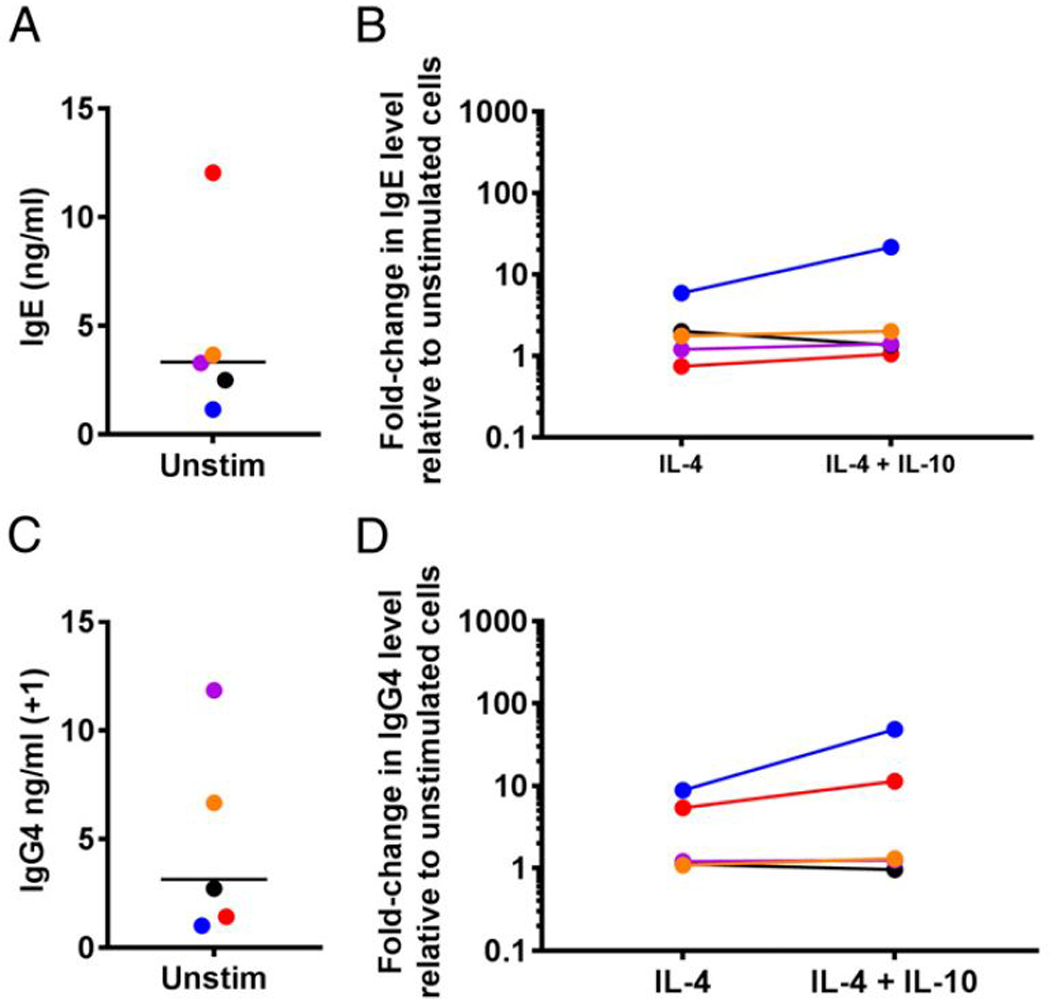
IL-4 and IL-10 do not significantly affect IgE and IgG4 production by PBMCs with STAT3 loss-of-function defects. (**A** and **C**) Spontaneous production of IgE (A) and IgG4 (C) by HIES PBMCs. (**B** and **D**) Fold change in levels of IgE (B) and IgG4 (D) in culture supernatants of HIES PBMCs stimulated with IL-4 with and without IL-10 relative to PBMCs cultured in media alone (*n* = 5). Each individual is represented with a unique color; colors are consistent for (A)–(D). Unstim, unstimulated.

To assess whether the effect of IL-10 on IL-4–induced IgE production involved signaling through the IL-10R, PBMCs from individuals with AD-HIES who have loss of junction STAT3 mutations (24, 26, 27) were stimulated with IL-4 and IL-10. Spontaneous production of IgE from AD-HIES PBMCs ranged from 1.14 ng/ml to 12.04 ng/ml with a GM of 3.33 ng/ml (Fig. 5A). Production of IgE with IL-10 alone was not significantly different from spontaneous production (data not shown). Following IL-4 stimulation, IgE production from AD-HIES PBMCs increased 2-fold and 6-fold over baseline in two donors and was minimally increased from baseline in the other three donors (Fig. 5B). Addition of IL-10 further increased IgE production in one donor who responded to IL-4 stimulation but otherwise had little to no effect (Fig. 5B). Spontaneous production of IgG4 ranged from undetectable levels to 10.86 ng/ml with a GM of 2.14 ng/ml (Fig. 5C). Production of IgG4 with IL-10 alone was not significantly different from spontaneous production (data not shown). Stimulation with IL-4 increased IgG4 production from baseline in two donors (nearly 9-fold and 5-fold from baseline), but not in the others. Addition of IL-10 augmented IgG4 production in the same donors who responded to IL-4 stimulation, but had no effect on the other donors (Fig. 5C).

## DISCUSSION

In this work, we show that IL-10 indirectly downregulates IgE production and class switching through accessory cells present in PBMCs while acting directly on B cells to upregulate IgG4 production. We also examined the ability of IgE^+^ and IgG4^+^ B cells to respond to IL-10.

We found that IL-10 is able to act directly on B cells to upregulate IL-4-induced production of IgG4 (Fig. 1D), a finding consistent with a previous study (14). This effect appears, however, to be downstream of class switching, as IL-10 had no significant effect on the expression of IL-4-induced expression of Cγ4 GLTs in isolated B cells (Fig. 1F). The B cells primarily responsible for the upregulation of IgG4 production in response to IL-10 are CD27^+^ B cells (Fig. 2D), cells thought of as Ag-experienced. Indeed, IL-10R expression was most frequent in plasmablasts and memory cells (Fig. 3B), both of which are CD27^+^ and have generally an increased capacity for Ig production compared with transitional or naive B cells (29). IL-10 was also able to directly enhance the IL-4–induced production of other Ig isotypes (see Supplemental Fig. 3) with the exception of IgE. This is not due to an inability of IgE-producingB cells to respond to IL-10, because the frequency of IL-10R expression is similar between IgE^+^ and IgG4^+^ cells (Fig. 3B). However, it is possible that IL-10R function may somehow differ in IgE^+^B cells.

Studies examining the effect of IL-10 on isolated tonsillar and peripheral B cells have shown that IL-10 can upregulate or downregulate IgE production or have no effect (14,15, 30–32). This variability is reflected in our data (Figs. 1, 2) and suggests that another factor besides, or in addition to, IL-10 is needed to consistently downregulate IgE production. Indeed, we find that IL-10 is able to downregulate IgE production when B cells are in the context of other cells (Fig. 4).

Our findings that IL-10 decreases IL-4–induced IgE production by PBMCs is in agreement with other studies (14, 15), including studies in atopic individuals (14, 33). The lack of a consistent response to IL-10 from isolated B cells suggests that another cell is needed to effectively downregulate IL-4–induced IgE production. Studies have suggested that monocytes maybe required (15), although others have suggested that T cells are necessary (14, 33).

We found that IL-10 did not have a consistent effect on IL-4–induced IgG4 production by PBMCs (Fig. 4D), a finding reflected in differing conclusions by previous studies examining the effect of IL-10 on IgG4 production (14,15,18). Differences seen among these various studies may be related to differences in anti-CD40 Abs or in the concentrations used or the use of CD40L rather than anti-CD40. Although we did not see a difference in the effect of IL-10 with the use of CD40L compared with anti-CD40 at different concentrations (data not shown), there is evidence that different concentrations of anti-CD40 or CD40L have dose-dependent effects on IgE and IgG4 production (5,14).

Following binding of IL-10 to the IL-10R α-chain, conformational changes allow the recruitment of IL-10R β-chains and the association of Jak1 and Tyk2. Phosphorylation of IL-10Rα by these kinases allows recruitment of STAT3, which is then phosphorylated and moves to the nucleus (22). Patients with AD-HIES have dominant-negative mutations in STAT3 and highly elevated levels of serum IgE (24,26,27). One mechanism by which these patients acquire elevated IgE levels could be due to impaired IL-10R signaling such that they are unable to respond to normal inhibitory signals for IgE production normally provided by IL-10. However, we were unable to show any effect of IL-10 on IL-4–induced IgE production because of a relative lack of upregulation of IgE production by HIES PMBCs by IL-4 (Fig. 5). Lack of response to IL-4 with regards to IgE and IgG4 production by HIES cells has been seen in previous studies (34, 35), and our data further the notion that B cells from HIES patients are maximally producing IgE and IgG4 (35). Interestingly, in the HIES donors responding to IL-4, the addition of IL-10 had no effect in one and markedly improved IgE production in the other (Fig. 5A), raising the possibility of an abnormal response to IL-10 in these donors; however, no conclusions can be made. Whether IL-10 has a downregulatory effect on IgE production by HIES cells that is induced by other cytokines (35) remains to be seen and is the focus of future work.

It has been shown recently that TLR9 ligation inhibits class switch to IgE and IgE production in human B cells (36). This inhibition is dependent on STAT3, which is linked to TLR9 signaling through dedicator of cytokinesis 8 (DOCK8) (4, 36). Interestingly, TLR9 ligands also stimulate IL-10 production by B cells (21). Whether TLR9 induction of B cell IL-10 production and subsequent autocrine or paracrine signaling play a role in downregulation of IgE production by TLR9 ligation is not known.

Taken together, our results suggest that in the presence of IL-10 (i.e., an allergen-tolerizing environment), IL-10 acts through accessory cells to inhibit class switch to, and production of, IgE. In this same tolerizing environment, IL-10 may act directly on Ag- experienced, switched B cells to promote production of IgG4 (and other Ig isotypes except IgE); this direct action of IL-10 is likely downstream of germline transcription. The indirect effects of IL-10 on Cε GLT expression and IgE production are applicable to unswitched B cells, which do not frequently express IL-10R, that enter lymphoid tissue to encounter a milieu of other cells guiding their class switch toward or away from IgE. However, in tissues, where more differentiated, switched B cells with a higher frequency of IL-10R expression are found, IL-10 acts directly to enhance production of IgG4. These two effects of IL-10 result in increased IgG4/IgE ratios that have been shown to alter allergic effector responses. Further study of the cells and mechanisms involved in IL-10’s downregulation of IgE class switch and production and of the direct enhancement of IgG4 production will lead to better understanding of and treatment modalities for allergic diseases.

## Supplementary Material

Suppl

## Abbreviations used in this article:

AD-HIES: autosomal dominant hyper-IgE syndrome
BD: BD Biosciences
GLT: germline transcript
GM: geometric mean
HIES: hyper-IgE syndrome
MB: Miltenyi Biotec
TFS: Thermo Fisher Scientific
