# Supplementary material for: IL-10 Indirectly Downregulates IL-4–Induced IgE Production by Human B Cells
Source: Immunohorizons. Author manuscript; Available in PMC 2022 Mar 2. (PMC8890443; doi:10.4049/immunohorizons.1800076)
Supplement: Suppl [file NIHMS1056838-supplement-Suppl.pdf]

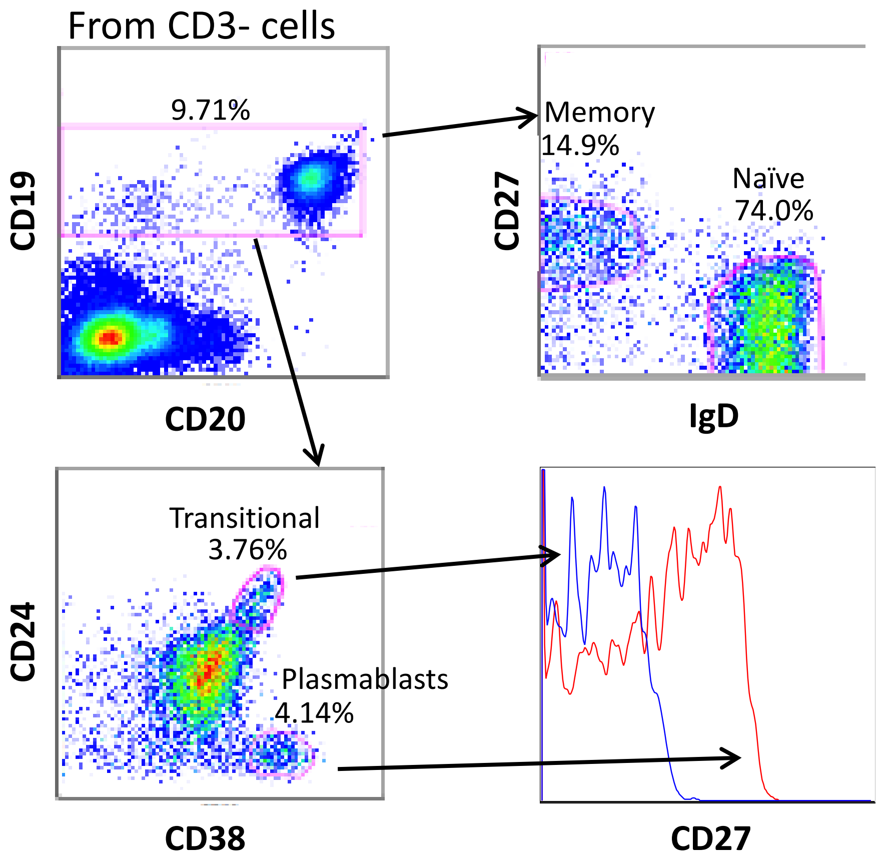

SUPPLEMENTAL FIGURE 1. Gating strategy and staining for B cell subsets. Cells expressing CD3 were first excluded; of the remaining cells, CD19+ cells were selected before examining staining for CD24 and CD38 (transitional and plasmablast subsets), and CD27 and IgD (naïve and memory subsets).

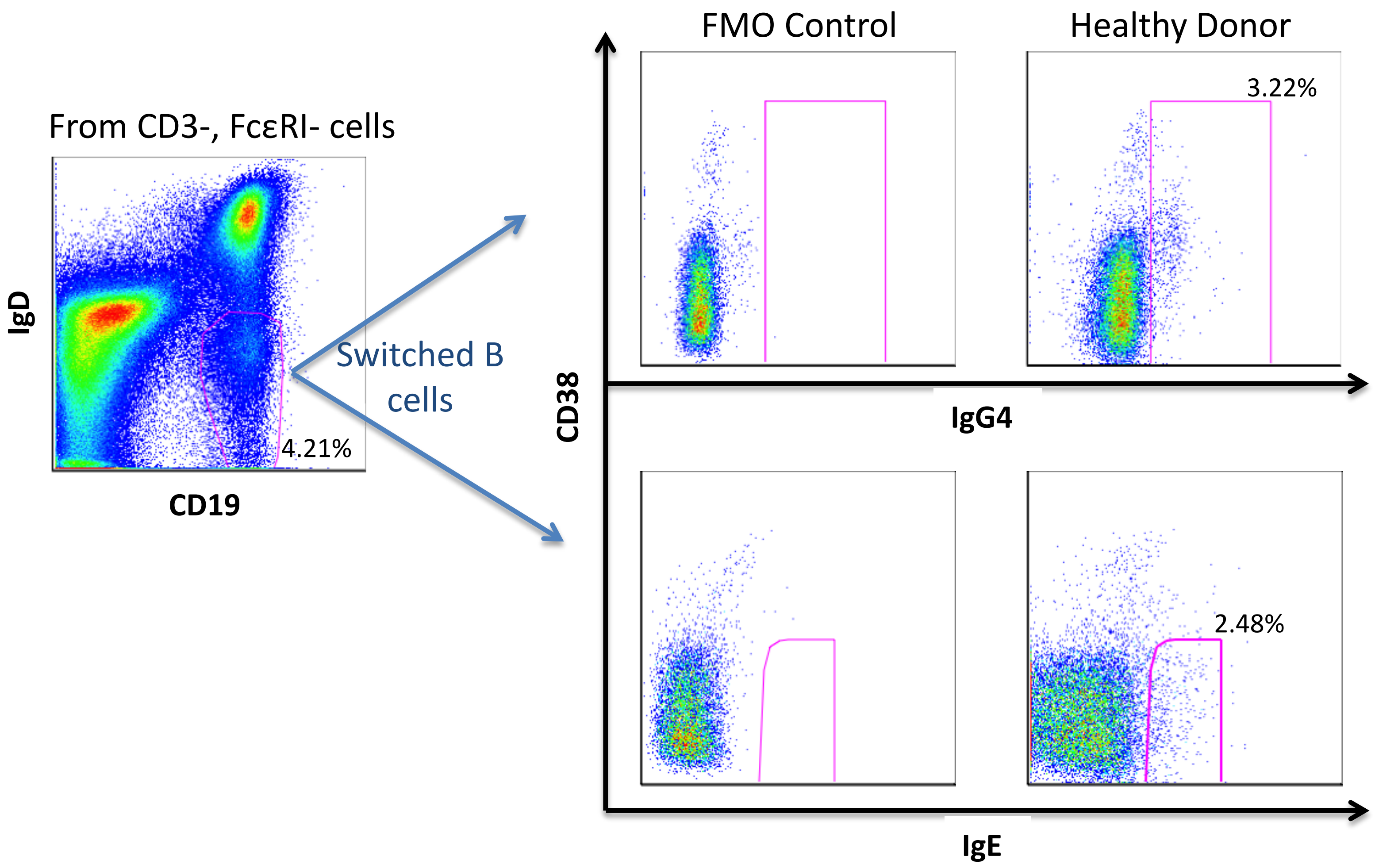

SUPPLEMENTAL FIGURE 2. Gating strategy and staining for IgE+ and IgG4+ B cells. Cells expressing CD3 and FcεRI were first excluded; of the remaining cells, CD19+ IgD- cells were selected before examining staining for IgE and IgG4. Left panels denote fluorescence minus one (FMO) controls for IgG4 and IgE. Right panels show staining in unstimulated cells.

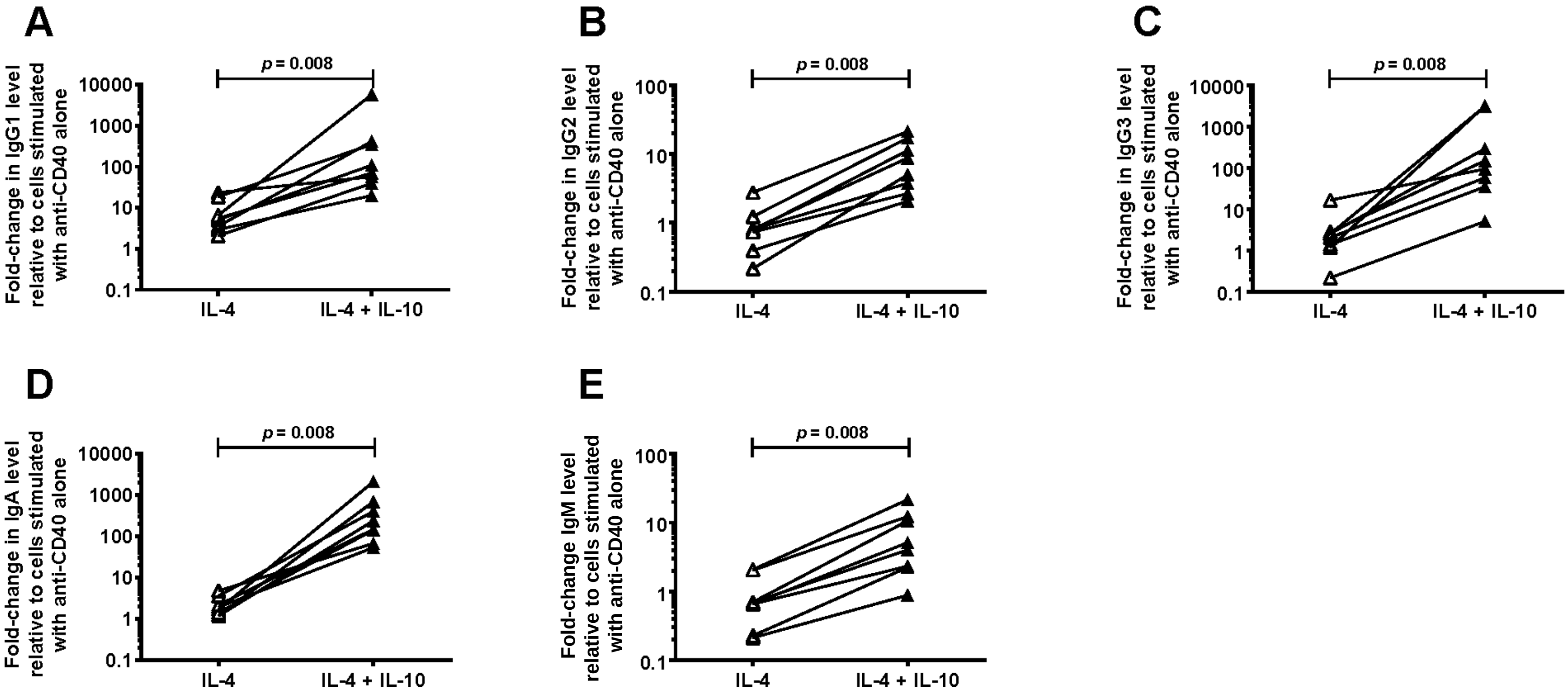

SUPPLEMENTAL FIGURE 3. IL-10 enhances the IL-4-induced production of non-IgE isotypes. Fold change in levels of IgG1 (**A**), IgG2 (**B**), IgG3 (**C**), IgA (**D**), and IgM (**E**) in culture supernatants of isolated total B cells stimulated with anti-CD40 and IL-4 with (filled triangles) and without (open triangles) IL-10, relative to B cells cultured with anti-CD40 antibody alone. $n = 8$. p values were determined using Wilcoxon matched-pairs signed rank test.
